# Methods to estimate baseline creatinine and define acute kidney injury in lean Ugandan children with severe malaria: a prospective cohort study

**DOI:** 10.1186/s12882-020-02076-1

**Published:** 2020-09-29

**Authors:** Anthony Batte, Michelle C. Starr, Andrew L. Schwaderer, Robert O. Opoka, Ruth Namazzi, Erika S. Phelps Nishiguchi, John M. Ssenkusu, Chandy C. John, Andrea L. Conroy

**Affiliations:** 1 Child Health and Development Center, Makerere University College of Health Sciences, Kampala, Uganda; 2 Department of Pediatrics, Division of Nephrology, Indiana University School of Medicine, Indianapolis, Indiana USA; 3 Department of Paediatrics and Child Health, Makerere University College of Health Sciences, Kampala, Uganda; 4 Developmental Behavioral Pediatrics, University of Washington, Seattle, Washington USA; 5 Department of Epidemiology and Biostatistics, Makerere University School of Public Health, Kampala, Uganda; 6 Department of Pediatrics, Ryan White Center for Pediatric Infectious Disease and Global Health, Indiana University School of Medicine, 1044 W. Walnut St., Indianapolis, IN 46202 USA

**Keywords:** Acute kidney injury, Baseline creatinine, Schwartz, Pottel, Severe malaria, Sub-Saharan Africa, Methods, Mortality, Undernutrition, Pediatric

## Abstract

**Background:**

Acute kidney injury (AKI) is increasingly recognized as a consequential clinical complication in children with severe malaria. However, approaches to estimate baseline creatinine (bSCr) are not standardized in this unique patient population. Prior to wide-spread utilization, bSCr estimation methods need to be evaluated in many populations, particularly in children from low-income countries.

**Methods:**

We evaluated six methods to estimate bSCr in Ugandan children aged 6 months to 12 years of age in two cohorts of children with severe malaria (*n* = 1078) and healthy community children (*n* = 289). Using isotope dilution mass spectrometry (IDMS)-traceable creatinine measures from community children, we evaluated the bias, accuracy and precision of estimating bSCr using height-dependent and height-independent estimated glomerular filtration (eGFR) equations to back-calculate bSCr or estimating bSCr directly using published or population-specific norms.

**Results:**

We compared methods to estimate bSCr in healthy community children against the IDMS-traceable SCr measure. The Pottel-age based equation, assuming a normal GFR of 120 mL/min per 1.73m^2^, was the more accurate method with minimal bias when compared to the Schwartz height-based equation. Using the different bSCr estimates, we demonstrated the prevalence of KDIGO-defined AKI in children with severe malaria ranged from 15.6–43.4%. The lowest estimate was derived using population upper levels of normal and the highest estimate was derived using the mean GFR of the community children (137 mL/min per 1.73m^2^) to back-calculate the bSCr. Irrespective of approach, AKI was strongly associated with mortality with a step-wise increase in mortality across AKI stages (*p* < 0.0001 for all). AKI defined using the Pottel-age based equation to estimate bSCr showed the strongest relationship with mortality with a risk ratio of 5.13 (95% CI 3.03–8.68) adjusting for child age and sex.

**Conclusions:**

We recommend using height-independent age-based approaches to estimate bSCr in hospitalized children in sub-Saharan Africa due to challenges in accurate height measurements and undernutrition which may impact bSCr estimates. In this population the Pottel-age based GFR estimating equation obtained comparable bSCr estimates to population-based estimates in healthy children.

## Background

Globally, 1.7 million people die from acute kidney injury (AKI) every year and 80% of deaths occur in low-and middle-income countries (LMIC) [1]. Mortality from AKI in children in sub-Saharan Africa is 34% compared to a global average of 14% [2]. In contrast to high-income countries where the majority of AKI is hospital-acquired and managed in intensive care settings, the majority of AKI in LMIC is community-acquired [3]. As such, using admission creatinine to estimate baseline SCr (bSCr) likely leads to AKI under-recognition. In a study of Ugandan children with severe malaria with serial creatinine measures, half had their peak creatinine measured on admission [4].

AKI is diagnosed by increased serum creatinine or decreased urine output [5]. Accurate diagnosis of AKI relies on knowing bSCr which is ideally measured within 3 months of hospital admission. However, bSCr values are often unavailable in hospitalized patients, requiring bSCr estimation. A number of studies have evaluated approaches to estimate bSCr in pediatric populations in high-income countries [6–8]. The most commonly used approach is to assume a normal estimated glomerular filtration rate (eGFR) of 120 mL/min per 1.73m^2^ and use the Bedside Schwartz equation to back-calculate bSCr [6, 8–10]. This approach has been used in large multi-national studies [10], but has not been validated in LMIC settings where there are limited population-based estimates of creatinine in children.

Creatinine is affected by non-renal factors including muscle mass and nutritional status [11]. It is important to validate approaches to estimate bSCr in LMIC where undernutrition is more prevalent and lower estimated bSCr relative to height due to less muscle mass per body surface area may result in eGFR being overestimated [12]. Another challenge in using the bedside Schwartz equation to back-calculate estimated bSCr is the requirement for a height measurement. Height measurements are not always available in LMIC and it can be challenging to accurately measure height in the context of critical illness. As such, the development of validated height-independent approaches to estimate bSCr in LMIC would simplify AKI diagnosis. Recently, there have been efforts to measure creatinine and evaluate the burden of kidney disease in adults in sub-Saharan Africa [13–16], but data from pediatric populations are lacking [17].

In this study we evaluate six methods to estimate bSCr in two cohorts of Ugandan children. We evaluated the accuracy, bias and precision of methods to estimate bSCr compared to measured values in healthy children. In children with severe malaria, we evaluated the prevalence of AKI, and the relationship between AKI and mortality. We hypothesize that standard methods to define bSCr may lead to the underestimation of AKI in LMIC settings.

## Methods

### Study populations

**Fig. 1:**
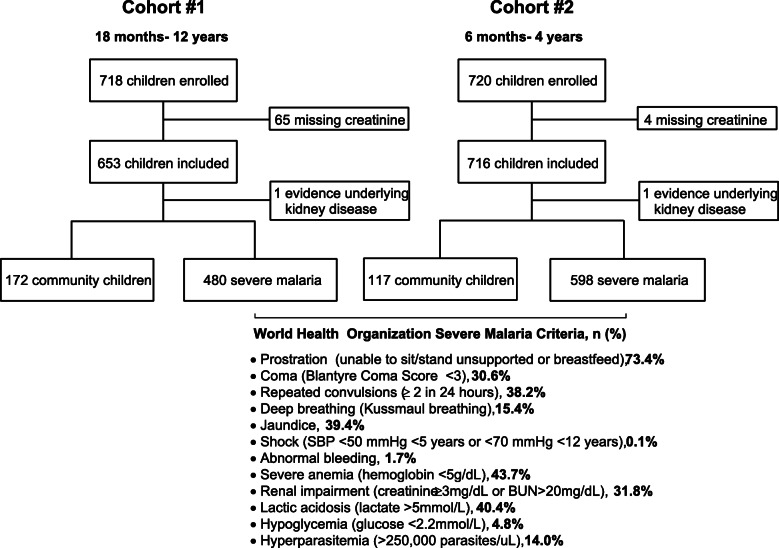
Flow chart of the two study populations. Between 2008 and 2017, 289 community children and 1078 children with severe malaria were enrolled in two severe malaria studies that recruited children from Kampala and Jinja in Uganda. One community child from each cohort with evidence of underlying kidney disease (estimated GFR < 90 mL/min per 1.73m^2^) was excluded from the study

We used two severe malaria cohorts that prospectively recruited children in Uganda between 2008 and 2017, and a population of healthy community children as controls (Fig. 1). All children with severe malaria had *P. falciparum* on blood smear or a positive rapid diagnostic test for *Plasmodium falciparum* HRP-2, and met World Health Organization criteria for severe malaria [18]. Severe malaria was defined according to WHO criteria: coma (Blantyre Coma Score < 3), deep breathing, multiple convulsions, prostration, shock, abnormal bleeding, and jaundice; and laboratory indices of disease severity (severe anemia, hypoglycemia, hyperlactatemia, hyperparasitemia) (Fig. 1) [18]. Cohort 1 enrolled children meeting specific case definitions for cerebral malaria or severe malarial anemia [19]. Cohort 1 enrolled children aged 18 months to 12 years of age between 2008 and 2013 from Mulago National Referral Hospital in Kampala, Uganda [19]. Cohort 2 enrolled children with a broad range of severe malaria criteria: cerebral malaria, respiratory distress, multiple seizures, severe malarial anemia, or prostration. Cohort 2 enrolled children 6 months to 4 years of age between 2014 and 2017 from Mulago National Referral Hospital in Kampala or Jinja Regional Referral Hospital in Jinja, Uganda. Although children were enrolled in each study on the basis of specific severe malaria criteria, multi-organ dysfunction was common and children often presented with multiple severe malaria criteria (Fig. 1). Exclusion criteria included known chronic illness requiring medical care, known developmental delay, or history of coma, head trauma, hospitalization for malnutrition, or cerebral palsy. Additional exclusion criteria for the community children (CC) included any illness requiring medical care within the previous 4 weeks or major medical or neurologic abnormalities at screening physical examination, including a known history of kidney and urinary tract birth defects, or chronic renal disease. Community children with an eGFR < 90 mL/min per 1.73m^2^ calculated from the enrollment serum creatinine were excluded (*n* = 2). Community children in both studies were recruited from the nuclear family, extended family, or household area of children with severe malaria.

At enrollment all children had a history and physical exam and had blood drawn for clinical tests and EDTA-anticoagulated plasma stored at − 80 °C until testing. Children were weighed on a standardized electronic weighing scale. Height was measured using a stadiometer (recumbent) or a wall-mounted tape measure (standing height). Recumbent height was taken on all children with SM at enrollment and standing at follow-up visits, while the community children were assessed standing for all visits. For cohort 2, the recumbent length was taken on all children across visits. Heights and weights were converted into z scores (height-for-age, weight-for-age, weight-for-height or bmi-for-age) based on 2006 World Health Organization (WHO) or 2007 WHO growth references based on age taking into consideration whether the measure was taken standing or recumbent [20, 21]. Undernutrition was defined as a z score < − 2 as follows: underweight (weight-for-age), stunting (height-for-age), or wasting (weight-for-height or BMI-for-age). Children were tested for sickle cell anemia as described [22]. A single blood pressure reading was taken on enrollment for all children using available instruments. As categorization of blood pressure based on standardized percentiles requires three independent measurements [23], we do not present data on hypertension in the population.

### Quality review of height measurements

In both cohorts serial heights were measured over follow-up. Children from cohort 1 had scheduled follow-up visits at 6, 12 and 24 months, and children from cohort 2 had scheduled follow-up visits at 1 month and 12 months. Heights were also measured on unscheduled sick visits over follow-up. To identify within-subject height outliers, linear mixed effects models were used and standardized residuals were used to flag heights for further review [24, 25]. Models included height as the dependent variable and age and sex as fixed effects with a subject specific random intercept and slope. Cohort 1 flagged children with a standardized residual > 2 or < − 2 and cohort 2 used more stringent criteria of > 1 or < − 1 as more frequent height assessments were available for comparison. Heights flagged were independently reviewed for trend over time by two pediatricians (EPN and AB) using height-for-age and weight-for-age z scores over time to identify outliers and make adjustments consistent with the child’s individual growth trajectory (*n* = 22 adjustments, cohort 1; *n* = 15 adjustments, cohort 2). If heights were missing on admission, heights recorded at follow-up visits were used to interpolate the missing enrollment values (*n* = 5, cohort 1; *n* = 7, cohort 2). If height was missing and a follow-up height was not available (e.g. death), missing heights were estimated using WHO height-for-age reference curves stratified by sex (*n* = 24) [20, 21]. When the heights for both cohorts were plotted on WHO height-for-age reference curves, the curve for the population corresponded to the 10th percentile. In instances admission heights were missing without a follow-up height available, children were assigned a height based on the 10th percentile for the WHO curve (*n* = 19, cohort 1; *n* = 25, cohort 2). Admission heights when children were ill were noted as the most difficult heights to accurately measure.

### Assessing kidney function

Creatinine was tested on cryopreserved enrollment samples using a Beckman Coulter AU680 using the modified Jaffe colorimetric method (Indiana University, Pathology Laboratory). Samples were sent on dry ice to the research laboratory at Indiana University, and then stored again at -80C until sent to the Indiana University Pathology clinical laboratory for creatinine testing between 2012 and 2014 (cohort 1) and 2018 (cohort 2). Acute kidney injury was defined using the Kidney Disease: Improving Global Outcomes (KDIGO) criteria [5]. AKI was defined as a 1.5-fold increase in creatinine over estimated baseline and was staged: stage 1, 1.5–1.9x increase in creatinine over baseline; stage 2, 2.0–2.9x increase over baseline; stage 3, ≥3.0x increase over baseline. A single creatinine measure was available on admission and data on urine output were not collected. Dialysis was not available on site at the time the study was conducted.

### Methods of estimating bSCr

**Fig. 2:**
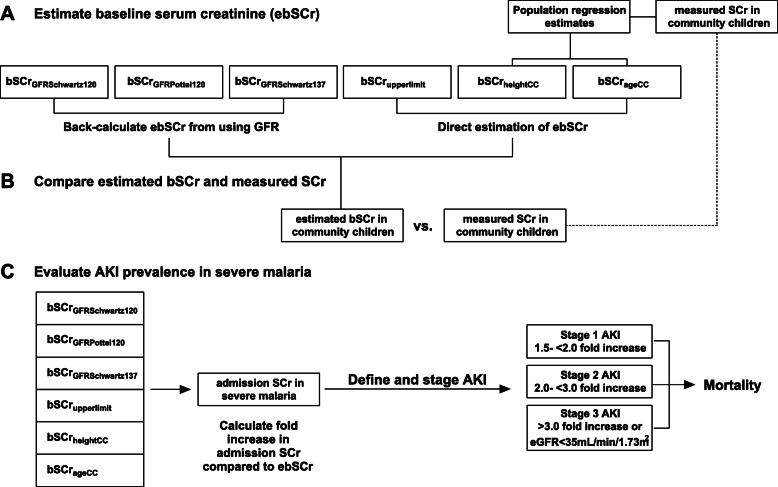
Overview of approaches used to evaluate estimates of baseline serum creatinine. **a** Overview of GFR-based and direct SCr-based methods to estimate baseline serum creatinine (bSCr) in community children. Linear regression models using the height and age of community children were used for direct estimation of ebSCr_heightCC_ and ebSCr_ageCC_. **b** The estimated bSCr and measured SCr were compared in community children to evaluate the bias, precision and accuracy of methods. **c** AKI was defined in children with severe malaria using the different approaches to estimate bSCr, and the relationship between AKI and mortality was evaluated

Six methods of estimating bSCr were evaluated (Fig. 2). First, we back-calculated estimated bSCr using an assumed eGFR value and height-dependent (Bedside Schwartz [26]) or height-independent (Pottel [27]) equations as shown below.

1. bSCr_GFRSchwartz120_ (AKI_Schwartz120_): back-calculate estimated bSCr using the Bedside Schwartz equation, where eGFR = (0.413*height)/SCr, assuming a normal GFR of 120 mL/min per 1.73m^2^ (height-dependent, reference).
2. bSCr_GFRPottel120_ (AKI_Pottel120_): back-calculate estimated bSCr using the Pottel age-based equation, where eGFR = 107.3/(SCr/Q), assuming a normal GFR of 120 mL/min per 1.73m^2^ (height-independent). Q = 0.0270*age + 0.2329.
3. bSCr_GFRSchwartz137_ (AKI_Schwartz137_): back-calculate bSCr using the Bedside Schwartz equation, where eGFR = (0.413*height)/SCr, assuming a normal GFR of 137 mL/min per 1.73m^2^ corresponding to the population mean for healthy children.

We then used non-GFR based approaches to estimate bSCr directly.

4. bSCr_upperlimit_: Estimate bSCr using published upper limits of normal by age category (height-independent) [28].
5. bSCr_heightCC_: Estimate bSCr using linear regression models to estimate bSCr based on height-for-creatinine data from the community children, where y = 0.00276*height + 0.03607.
6. bSCr_ageCC_: Estimate bSCr using linear regression models to estimate bSCr based on age-for-creatinine data from the community children, where y = 0.02058*age + 0.2199.

Population-derived regression models were developed in healthy community children (*n* = 239) without sickle cell trait (HbAS, *n* = 40) or disease (HbSS, *n* = 1). See Additional File for Figure S1 showing the regression models for height- and age-based estimation of bSCr using the community children.

### Role of the funding source

The funders had no role in the study design, analysis or decision to publish.

### Statistical analysis

Data were analyzed using STATA v14.0 (StataCorp) and GraphPad Prism v7.03. Differences between continuous measures were analyzed using Student’s t-test or one-way ANOVA, and differences in proportions were analyzed using Pearson’s Chi Square test or Fisher’s exact test, as appropriate. To evaluate differences in the measured vs. estimated baseline creatinine in community children, Bland-Altman plots were generated. To compare the difference between the measured and estimated bSCr, we used a paired Wilcoxon signed rank test. Bias was represented by the total mean difference between estimated and measured creatinine, and compared to a hypothetical value of 0 using a one-sample t-test. Precision represents one standard deviation of the bias. Proportional bias was evaluated by testing if the slope of the linear regression model of the differences between estimated and measured creatinine against the average of estimated and measured creatinine differed from zero [29]. To evaluate accuracy, we compared the percentage of estimated bSCr values that fell within 10% (p10) or 30% (p30) of the measured creatinine. Binomial exact confidence intervals were used to estimate the 95% confidence estimates for the AKI prevalence. To evaluate the relationship between AKI and mortality we used generalized linear models to estimate the risk ratio (RR) using binomial family and log link adjusting for age and sex. Model fit was assessed using Akaike information criterion (AIC) and Bayesian information criterion (BIC). Receiver operating characteristic (ROC) curves were also used to evaluate the relationship between AKI and mortality. A *p* value < 0.05 was considered significant.

## Results

The present study prospectively enrolled children from 2008 to 2013 (Cohort # 1, 18 months to 12 years of age) and 2014–2018 (Cohort #2, 6 months to 4 years of age) (Fig. 1). The mean age (SD) of community children was 3.3 (1.9) years compared to 2.8 (1.6) years in children with severe malaria (Table 1). There was a sex difference in community children compared to children with severe malaria with 51.2% of community children female compared to 42.2% of children with severe malaria. There was no difference in height-for-age z scores between the community children and children with severe malaria. Children with severe malaria had lower weight-for-age and weight-for-height z scores and were more likely to be underweight or wasted compared to community children (Table 1). Overall, 24.7% of children were stunted, 17.7% underweight and 12.3% wasted.

**Table 1:** Demographic characteristics of study population

	Community children (CC)			Severe Malaria (*n* = 1078)	*P* value Combined CC vs. Severe Malaria
	Cohort #1 (*n* = 172)	Cohort #2 (*n* = 117)	Combined (*n* = 289)		
Enrollment years	2008–2013	2014–2017	2008–2017	2008–2017	–
Age, years					
Mean (SD)	4.01 (2.03)	2.22 (1.02)	3.28 (1.91)	2.83 (1.62)	0.0001
Median (IQR)	3.59 (2.61, 4.62)	2.21 (1.39, 3.09)	3.00 (2.06, 3.92)	2.50 (1.75, 3.47)	
Min, Max	1.55, 11.44	0.53, 3.96	0.53, 11.44	0.46, 11.69	
Age category, n(%)					
< 1	0 (0.0)	18 (15.4)	18 (6.2)	74 (6.9)	0.001
1- < 2	11 (6.4)	35 (29.9)	46 (15.9)	296 (27.5)	
2–5	128 (74.4)	64 (54.7)	192 (66.4)	609 (56.5)	
> 5	33 (19.2)	0 (0.0)	33 (11.4)	99 (9.2)	
Sex, % F	94 (54.7)	55 (47.0)	149 (51.2)	455 (42.2)	0.004
Height, cm	96.6 (13.6)	83.7 (10.4)	91.4 (13.9)	88.8 (12.8)	0.0032
Weight, kg	14.7 (4.4)	11.6 (2.6)	13.5 (4.1)	12.0 (3.6)	< 0.0001
BMI, kg/m^2^	15.6 (1.7)	16.5 (1.7)	15.9 (1.7)	15.1 (1.8)	< 0.0001
Weight-for-age^a^	−0.82 (0.98)	− 0.47 (1.08)	− 0.68 (1.04)	−1.10 (1.10)	< 0.0001
Underweight^a^ n (%)	21 (12.5)	9 (7.7)	30 (10.5)	209 (19.6)	< 0.0001
Height-for-age^b^	−1.25 (1.13)	−1.26 (1.34)	− 1.25 (1.22)	−1.17 (1.35)	0.3602
Stunted, n (%)	43 (25.0)	36 (30.8)	79 (27.3)	259 (24.1)	0.250
Weight-for-height < 5^c^	−0.15 (1.25)	0.30 (1.02)	0.05 (1.17)	−0.65 (1.32)	< 0.0001
BMI-for-age > 5^d^	−0.30 (0.84)	–	−0.30 (0.84)	− 0.80 (1.43)	0.0635
Wasted, n (%)^e^	8 (4.7)	3 (2.6)	11 (3.8)	156 (14.6)	< 0.0001
Systolic BP	94.1 (10.2)	98.5 (11.7)	95.9 (11.1)	95.9 (13.0)	0.9164
Diastolic BP	60.9 (10.6)	59.1 (9.8)	60.2 (10.4)	56.9 (12.1)	< 0.0001
Enrollment SCr					
Mean ± SD	0.31 (0.08)	0.26 (0.07)	0.29 (0.08)	0.48 (0.48)	< 0.0001
Median (IQR)	0.30 (0.24, 0.35)	0.25 (0.20, 0.30)	0.28 (0.23, 0.33)	0.38 (0.29, 0.49)	
Min, Max^a^	0.19, 0.56	0.19, 0.46	0.19, 1.0	0.19, 7.3	
Enrollment eGFR, ml/min per 1.73m^2^	136.4 (29.3)	137.7 (30.5)	136.9 (29.8)	99.5 (38.2)	< 0.0001

Data are presented as mean (SD) unless otherwise indicated. Undernutrition (weight-for-age z score < −2), stunted (height-for-age z score < −2), wasted (weight-for-height or bmi-for-age z score < −2) according to WHO 2006 (age 0–5 years) and 2007 (5–12 years) reference standards

^a^Weight-for-age z scores available for children < 10 years (Cohort #1, CC *n* = 168, SM *n* = 476; Cohort #2, CC *n* = 117, SM n = 591; Combined, CC *n* = 285; SM, *n* = 1067)

^b^Height-for-age z scores (Cohort #1, CC *n* = 168, SM *n* = 479; Cohort #2, CC *n* = 117, SM *n* = 598; Combined, CC *n* = 289; SM, *n* = 1077)

^c^Weight-for-height z scores available for children < 5 years (Cohort #1, CC *n* = 139, SM *n* = 388; Cohort #2, CC *n* = 117, SM *n* = 591; Combined, CC *n* = 256; SM, *n* = 979)

^d^BMI-for-age z scores available for children ≥5 years of age (Cohort #1, CC *n* = 33, SM = 91; Cohort #2, CC *n* = 0, SM *n* = 0; Combined, CC *n* = 33; SM, *n* = 91)

^e^Wasted (Cohort #1, CC *n* = 172, SM = 479; Cohort #2, CC *n* = 117, SM *n* = 591; Combined, CC *n* = 289; SM, *n* = 1070)

Nutritional status affects creatinine levels and malnourished children tend to have lower creatinine levels and overestimate GFR [30]. When we calculated eGFR using measured creatinine levels and the bedside Schwartz equation in community children, the mean eGFR in community children was 137 mL/min per 1.73m^2^, which is higher than the assumed normal GFR of 120 mL/min per 1.73m^2^ that is used as assumed baseline in pediatric AKI research [6, 8, 10].

The prevalence of sickle cell trait in Ugandan children is estimated to be 13.3% and sickle cell disease 0.7% [31]. There were 49 (16.5%) community children with sickle cell trait (HbAS) and one (0.3%) with sickle cell anemia (HbSS). There were no differences in eGFR or measured SCr in the community children based on the presence of sickle cell trait (mean (SD): HbAA, 0.29 (0.08) vs. HbAS, 0.30 (0.07), *p* = 0.207; eGFR, HbAA, 137.7 (30.2) vs. HbAS, 132.5 (27.7), *p* = 0.265).

### Approaches to estimate creatinine in community children

**Fig. 3:**
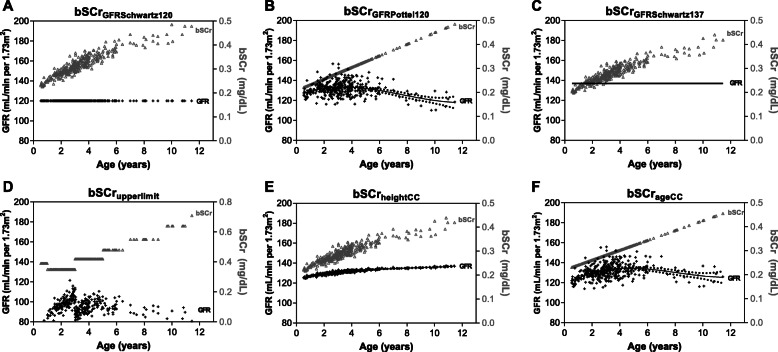
Estimated GFR and bSCr in healthy community children based on method to estimate bSCr. Graphs showing the GFR calculated off the estimated bSCr using the Bedside Schwartz equation on the left y-axis in black and the estimated bSCr in grey on the right y-axis. The top row represents approaches starting with an assumed GFR and back-calculating bSCr using either the Bedside Schwartz equation (**a**, **c**) or the Pottel height-independent equation (**b**). The bottom row represents approaches to estimate bSCr directly using the upper limit of normal (**d**) or height (**e**) and age (**f**) based estimates from community children. The eGFR for each graph was calculated using the Bedside Schwartz equation using the bSCr and height at enrollment, where eGFR = (0.413*height)/bSCr

**Fig. 4:**
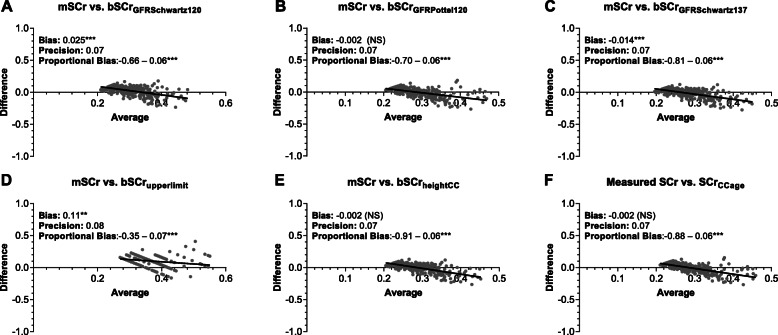
Bland Altman analysis comparing measured SCr in community children versus different methods to estimate bSCr. Graphs show the difference in estimated baseline SCr (bSCr) and measured (mSCr) compared to the average. The top row represents approaches starting with an assumed GFR and back-calculating bSCr using either the Bedside Schwartz equation (**a**, **c**) or the Pottel height-independent equation (**b**). The bottom row represents approaches to estimate bSCr directly using the upper limit of normal (**d**) or height (**e**) and age (**f**) based estimates from the community children. The bias represents the difference between the mSCr and estimated bSCr where the significance of bias was assessed using a one sample t-test comparing the mean bias to 0. The precision is represented as one standard deviation of the mean difference. Proportional bias was evaluated by testing if the slope of the linear regression model of the differences between estimated and measured creatinine against the average of estimated and measured creatinine differed from zero. Proportional bias represents the slope (B1) + SE (standard error) and the asterisks indicate whether the slope is statistically different from zero. Not significant (NS, where *p* > 0.05), *p < 0.05, **p < 0.001, ***p < 0.0001

To evaluate the most appropriate approach to estimate bSCr in this population (Fig. 2), we compared the measured and estimated baseline creatinine and eGFR in community children (Fig. 3). We evaluated the bias, precision and accuracy of the different approaches to estimate bSCr against the measured values in the community children using Bland-Altman analysis (Table 2, Fig. 4). When comparing the measured vs. estimated bSCr values using a paired Wilcoxon signed rank test, four methods had comparable measured and estimated bSCr values: the height (bSCr_heightCC_) and age-based (bSCr_ageCC_) linear regression estimates as well as bSCr_GFRSchwartz137_ and bSCr_GFRPottel120_. The same methods had the highest accuracy with the percentage values within 10% of the measured value ranging from 29 to 35% and the percentage of values within 30% of the measured value within 78–82% and three methods (bSCr_heightCC_, bSCr_ageCC_, bSCr_GFRPottel120_) did not show significant bias (Table 2, Fig. 4). Two approaches over-estimated bSCr: the upper limit of normal (bSCr_upperlimit_), and bSCr_GFRSchwartz120_, which is the current standard in pediatric AKI research. The estimated bSCr using the mean of the community children (bSCr_GFRSchwartz137_) was comparable to the measured value in the population, but underestimated bSCr in infants. All methods showed proportional bias in Bland-Altman analysis with a significant slope of the regression line comparing the difference and average between measured and estimated bSCr at higher creatinine values (Table 2, Fig. 4).

**Table 2:** Agreement between measured and estimated serum creatinine in community children

	Measured	Estimated					
		bSCr back-calculated from GFR			bSCr estimated directly		
	SCr	bSCr_GFRSchwartz120_ (reference)	bSCr_GFRPottel120_	bSCr_GFRSchwartz137_	bSCr_upperlimit_	bSCr_heightCC_	bSCr_ageCC_
Mean (SD) (mg/dL)	0.29 (0.08)	0.31 (0.05)	0.29 (0.05)	0.28 (0.04)	0.40 (0.06)	0.29 (0.04)	0.29 (0.04)
Median (IQR) (mg/dL)	0.28 (0.23, 0.33)	0.31 (0.28, 0.34)	0.28 (0.26, 0.30)	0.27 (0.25, 0.30)	0.39 (0.35, 0.42)	0.28 (0.26, 0.31)	0.28 (0.26, 0.30)
Wilcoxon^a^	–	***	NS	NS	***	NS	NS
Min, max (mg/dL)	0.19, 0.56	0.23, 0.34	0.22, 0.48	0.20, 0.44	0.35, 0.71	0.22, 0.44	0.23, 0.46
Correlation, SCr (r)	–	0.495	0.485	0.495	0.407	0.495	0.485
Bias (mg/dL)	–	0.025***	−0.002 (NS)	−0.014***	0.11***	−0.002 (NS)	−0.002 (NS)
Precision (mg/dL)	–	0.07	0.07	0.07	0.08	0.07	0.07
Accuracy							
P10, %	–	24%	35%	33%	8%	29%	35%
P30, %	–	71%	79%	82%	33%	80%	78%
Proportional Bias	–	−0.66 ± 0.06 ***	− 0.70 ± 0.06***	−0.81 ± 0.06***	−0.35 ± 0.07***	−0.91 ± 0.06***	−0.88 ± 0.06***

Standard deviation (SD), IQR: Inter-quartile range, r: Spearman’s rho, NS: Not significant, *p < 0.05, **p < 0.001, *** *p* < 0.0001

^a^Wilcoxon signed rank test (paired) for comparison of entire distribution with measured creatinine

Bias: mean difference between estimated and measured creatinine. To estimate fixed bias, we compared value of the difference to a value of 0 using a one sample t-test

Precision: one standard deviation of the Bias

Proportional bias: the slope of the regression line of the differences between estimated and measured creatinine against the average of estimated and measured creatinine. A slope of 0 means no proportional bias

Accuracy reflects the percentage of estimated SCr within 10 or 30% of the measured SCr, where accuracy = [(measured SCr- estimated SCr)/measured SCr] *100

*P* value is the significance of the deviation of the slope from 0. ***p < 0.0001

As there are physiological increases in GFR from birth to 2 years of age, using a single eGFR value across all ages may not be appropriate and age-related normative GFR values for children < 2 years of age have been used in previous studies [8]. When eGFR was calculated using the Bedside Schwartz equation from bSCr_GFRPottel120_ or directly from estimated bSCr (bSCr_upperlimit_, bSCr_heightCC_, bSCr_ageCC_) there was an increase in GFR across age groups with the lowest GFR in infants aged 6 months to < 1 year of age (Fig. 3, Table 3). This trend is consistent with age-related increases in eGFR and SCr across age groups (*p* < 0.0001, One-way ANOVA, Table 3, Fig. 3) with the largest difference observed in infants [32].

**Table 3:** Estimates of bSCr and eGFR in healthy community children based on age at enrollment

	Age < 1 years (***n*** = 18)		Age 1 to < 2 years (***n*** = 46)		Age 2 to < 5 years (***n*** = 192)		Age ≥ 5 years (***n*** = 33)		***P*** value^1^	
	bSCr	eGFR	bSCr	eGFR	bSCr	eGFR	bSCr	eGFR	SCr	eGFR
**Reference**										
Measured SCr	0.23 (0.04)	127.3 (20.8)	0.25 (0.06)	135.9 (29.0)	0.29 (0.07)	138.2 (30.8)	0.37 (0.08)	136.0 (28.7)	< 0.001	0.484
**SCr estimated by back calculating from an assumed GFR**										
bSCr_GFRSchwartz120_^a^	0.24 (0.01)	120 (0.0)	0.27 (0.01)	120 (0.0)	0.32 (0.03)	120 (0.0)	0.41 (0.04)	120 (0.0)	< 0.001	–
bSCr_GFRPottel120_^b^	0.23 (0.003)	126.8 (4.6)	0.25 (0.01)	131.9 (5.0)	0.29 (0.02)	132.7 (7.0)	0.39 (0.05)	126.8 (8.0)	< 0.001	< 0.001
bSCr_GFRSchwartz137_^a^	0.21 (0.009)	137 (0.0)	0.24 (0.01)	137 (0.0)	0.28 (0.02)	137 (0.0)	0.36 (0.03)	137 (0.0)	< 0.001	< 0.001
**SCr estimated directly**										
bSCr_upperlimit_^c^	0.39 (0.0)	73.3 (3.1)	0.35 (0.0)	92.9 (4.5)	0.39 (0.03)	97.5 (6.8)	0.54 (0.07)	90.8 (7.1)	< 0.001	< 0.001
bSCr_CCheight_^d^	0.23 (0.01)	125.9 (0.8)	0.25 (0.01)	128.4 (0.9)	0.29 (0.02)	131.0 (1.4)	0.36 (0.03)	134.7 (1.2)	< 0.001	< 0.001
bSCr_CCage_^e^	0.23 (0.003)	121.8 (4.5)	0.25 (0.01)	128.8 (5.0)	0.29 (0.02)	132.5 (7.2)	0.37 (0.04)	131.6 (7.2)	< 0.001	< 0.001

^1^*P* value calculated using ANOVA to evaluate mean differences across age categories

^a^Bedside Schwartz equation (eGFR = 0.413*height/SCr) used to back calculate creatinine assuming a normal of 120 mL/min/1.73m^2^ or 137 mL/min/1.73m^2^ (mean of the community children)

^b^Pottel equation (eGFR = 107.3/(SCr/Q), where Q = 0.0270*age + 0.2329) used to back calculate creatinine assuming a normal of 120 mL/min per 1.73m^2^

^c^Upper limit using Ceriotti et al., *Clin Chem* 2008 54:3

^d^Linear regression model of height vs. creatinine using healthy community children to estimate baseline creatinine from model

^e^Linear regression model of age vs. creatinine using healthy community children to estimate baseline creatinine from model

### AKI prevalence in children with severe malaria

**Fig. 5:**
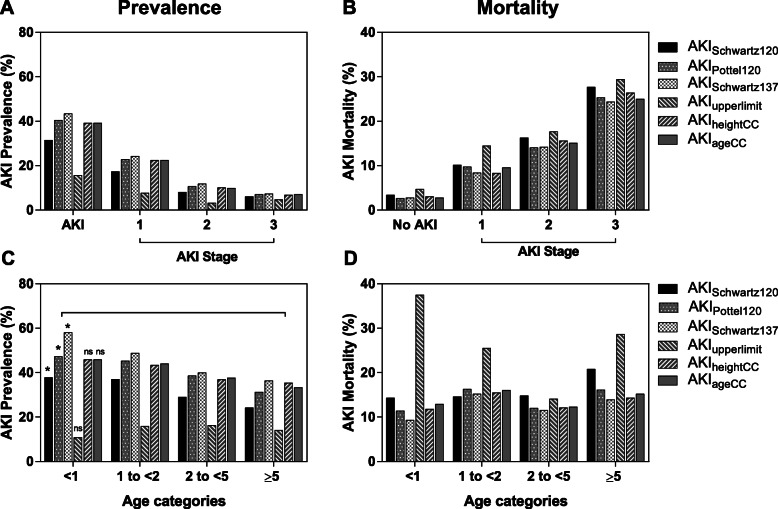
AKI prevalence and mortality across AKI stages and age categories in children with severe malaria. Bar graphs showing the frequency of AKI (**a**, **c**) or AKI mortality (**b**, **d**) using different approaches to estimate baseline serum creatinine (bSCr). **b** AKI severity was associated with increased mortality irrespective of method used to estimate bSCr (*p* < 0.0001 for all, Pearson’s Chi square test). **c** AKI prevalence differed across age categories when GFR based methods were used to estimate bSCr *p < 0.05. There was no difference in AKI prevalence when using approaches to directly estimate SCr (ns, *p* > 0.05). **d** AKI was associated with increased mortality across age categories

Using the different approaches to estimate bSCr we evaluated the prevalence of AKI on hospital admission in 1078 children with severe malaria (Figs. 2 and 5). AKI was based on a single admission creatinine and the six estimated bSCr values. AKI prevalence ranged from 15.6–43.4% (Fig. 5a, Additional file 1, Table S1). The majority of AKI was stage 1 with staging estimates ranging from: 7.7–24.3% Stage 1, 3.2–11.8% Stage 2, and 4.7–7.3% Stage 3 (Fig. 5a, Additional file 1, Table S1). The prevalence of AKI with the three approaches showing the best performance in estimating bSCr in the community children were as follows: i) AKI_GFRPottel120_, 40.4% (95% CI 37.5–43.3); ii) AKI_heightCC_, 39.2% (95% CI 36.3–42.2); iii) AKI_ageCC_, 39.2% (95% CI 36.3–42.2). Using the Bedside Schwartz equation assuming an eGFR of 120 mL/min per 1.73m^2^ underestimated AKI in this setting missing over 20% of AKI identified using population-based approaches (AKI_heightCC_, AKI_ageCC_). All approaches based on a fixed eGFR estimate (AKI_GFRSchwartz120_, AKI_GFRPottel120_, AKI_GFRSchwartz137_) resulted in an increase in AKI prevalence in infants aged 6 months to 1 year compared to the older age groups (Fig. 5c).

The Pottel equation had the highest area under the ROC curve, along with the highest sensitivity to predict mortality at 77.6%, and the highest risk ratio at 5.13 (95% CI 3.03–8.68) adjusting for child age and sex (Table 4). Mortality in children diagnosed with AKI using the Pottel equation was 13.6% overall and increased across AKI stages from 9.8% in stage 1 AKI, 14.0% in stage 2 AKI and 25.3% in stage 3 AKI. The relationship between AKI and mortality was consistent across age groups with change in mortality across age groups (Fig. 5d).

**Table 4:** Relationship between AKI and mortality

AKI Classification	Mortality no AKI N (%)	Mortality AKI N (%)	Risk Ratio (95% CI)	Model Fit		AKI Area under ROC		
				AIC	BIC		Sensitivity	Specificity
AKI_Schwartz120_	25 (3.4)	51 (15.1)	4.41 (2.78, 7.00)	0.473	− 6990.3	0.69 (0.64, 0.75)	67.1%	71.3%
AKI_Pottel120_	17 (2.7)	59 (13.6)	5.13 (3.03, 8.68)	0.469	− 6994.0	0.70 (0.65, 0.75)	77.6%	62.4%
AKI_Schwartz137_	17 (2.8)	59 (12.6)	4.50 (2.66, 7.62)	0.477	− 6986.2	0.68 (0.63, 0.73)	77.6%	59.2%
AKI_upperlimit_	43 (4.7)	33 (19.6)	4.17 (2.74, 6.36)	0.479	− 6984.1	0.65 (0.59, 0.71)	43.4%	86.5%
AKI_heightCC_	20 (3.1)	56 (13.3)	4.28 (2.61, 7.03)	0.477	−6986.3	0.69 (0.63, 0.74)	73.7%	63.4%
AKI_ageCC_	18 (2.8)	58 (13.7)	4.99 (2.98, 8.34)	0.470	− 6993.6	0.70 (0.65, 0.75)	76.3%	63.5%

Risk ratio estimated using a generalized linear model with binomial family and log link adjusting for age and sex

## Discussion

In the present study we evaluated six different approaches to estimate bSCr in Ugandan children. In this undernourished population of Ugandan children, assuming a normal GFR of 120 mL/min per 1.73m^2^ to calculate estimated bSCr underestimated AKI but retained good sensitivity to predict mortality. Of published methods, the Pottel height-independent approach to estimate bSCr in our population of lean Ugandan children was the most accurate and had the least bias. Further, the Pottel height-independent equation showed the strongest relationship with mortality in children with severe malaria.

There is a physiological increase in GFR from birth to age 2 [32]. A number of pediatric studies suggest using age-based normative eGFR values in children < 2 years of age to account for the physiological changes in GFR in this age group [7, 8]. In the present study we observed an increase in eGFR across age groups, but the eGFR was greater than 120 mL/min per 1.73m^2^ across all age groups, including infants, suggesting that using normative eGFR values in children < 2 years of age may not be as applicable in Ugandan children. This emphasizes the importance of nutritional status and body habitus when selecting methods to estimate bSCr and the need for evaluating and validating a range of equations. Efforts are underway to evaluate eGFR equations in adults across sub-Saharan Africa [15], and parallel efforts in children would be advantageous to better understand how measured GFR and estimated bSCr relate in this setting.

Our results are consistent with a high prevalence of AKI in children with severe malaria irrespective of age [25], and challenge the paradigm that AKI in severe malaria is more frequent in older children and adults [18]. The children in this study were 12 and under. Whether our results apply to older children who are more likely to have undergone puberty will need to be determined in future studies. We demonstrate that AKI is common across all age groups. There were no differences in mortality across age groups in this pediatric population. Larger studies will be needed to evaluate whether there are age-related differences in susceptibility to AKI and whether there are age-related differences in outcomes in pediatric populations.

The three approaches of back-calculating estimated bSCr based on a fixed eGFR showed differences in AKI prevalence across age groups with the highest prevalence seen in infants and the lowest prevalence observed in children < 5 years of age (Fig. 5c, Table S2). When comparing approaches of estimating bSCr in community children, bSCr estimates were closest to the measured value in infants. All approaches to estimate bSCr in the study population had a significant proportional bias as seen by a positive slope on the Bland-Altman plots in Fig. 4, and all models except the upper limit of normal underestimated bSCr in older children. As such, the prevalence of AKI in infants is likely accurate, but AKI may be underestimated in older children. In the present study the majority of the community children were < 5 years of age. A larger population of children > 5 years and adolescents are needed to establish norms across the pediatric age spectrum in this setting.

Accurate assessment of height in critically ill populations can be challenging, and appropriate measurement tools are not always available in resource-constrained settings. This study was able to assess the reliability of height measures over time using mixed effects models adjusting for child age and sex to correct individual-level outliers as described [24, 25]. We identified several heights that required adjustment based on the child’s individual growth trajectory or the mean height-for-age score for the population. Given potential limitations in obtaining accurate height measurements in critically ill populations, including infection-control concerns in certain high-risk populations (e.g. Ebolavirus [33]), the validation of age-based approaches to estimate bSCr in hospitalized patients in LMIC settings is essential.

The Pottel equation was derived from a large hospital database of normal creatinine in Belgian children and differs from the Bedside Schwartz equation, which was developed in children with chronic kidney disease. The height independent Pottel equation (eGFR = 107.3/(Scr/Q) uses age-based norms of creatinine defined by Q. Updates to the height-independent equation have been suggested to modify the calculation of Q by sex and age and extend it into adolescence and early adulthood (Hoste(age) [34]). The updated Hoste(age) equation and use of Q alone led to overestimation of creatinine in our study population. Thus, the original Pottel equation assuming a normal eGFR of 120 mL/min per 1.73m^2^ was the most appropriate in our population and closely resembled the linear regression estimates of bSCr_ageCC_ derived using measured values from community children. With the advantage of not requiring a height measurement, the Pottel age-based equation is more suitable for large population-based studies.

In this study we included the upper limit of normal to estimate AKI as this approach was used in the largest prospective study of AKI in Ugandan children that was conducted in 2055 hospitalized children aged 0–15 years at Mulago National Referral Hospital [28]. The prevalence of AKI in the study by Imani et al., was 13.5% overall, and was 5.4% in children with malaria [28]. In the present study the prevalence of AKI in children that met WHO severe malaria criteria on admission using the upper limit of normal was nearly three-fold higher at 15.6% compared to 5.4% in the study by Imani et al., and 40.4% using our recommended approach of AKI_Pottel120_. Therefore, we estimate that the prevalence of AKI in the study by Imani et al., would likely be between 27.2–35.0% using more sensitive approaches of bSCr_GFRSchwartz120_ or bSCr_GFRPottel120_ respectively. AKI is a well-established risk factor for mortality in critically ill populations. The ideal AKI definition should be sensitive as small increases in creatinine are associated with increased mortality [5, 10, 35]. Further, AKI is a risk factor for the long-term development of chronic kidney disease (CKD) in both adult and pediatric populations [25, 36–41]. In Ugandan severe malaria survivors AKI is a risk factor for long-term neurocognitive impairment and behavioral problems [25, 42]. The use of sensitive AKI definitions is important to identify children at risk of in-hospital mortality, but also to identify children at risk for post-discharge morbidity (e.g. repeated admissions, chronic kidney disease, cardiovascular disease), and mortality.

There were a number of limitations in this study. First, we were unable to directly measure GFR in the studied population using methodology such as iohexol clearance and had to rely on using equations developed in other contexts. None of the children with severe malaria had a known bSCr, so we had to evaluate approaches to estimate bSCr using healthy community children. As the methods to estimate bSCR were derived from the community children, this likely contributed to the good performance of the approaches. Additional prospective studies are required to externally validate these approaches in an independent cohort of Ugandan children. We were unable to rule out congenital malformations of the genitourinary system in the healthy children, so it is possible that children with clinically silent kidney disease were included. However, children with a history of chronic disease were not eligible for the study, and two children with evidence of underlying kidney disease were excluded from the study. It is possible some of the children with severe malaria had underlying kidney disease that made them susceptible to AKI and severe disease. However, as malaria is an acute febrile illness that typically occurs in previously healthy children, we expect the number of children with underlying kidney disease in the severe malaria group to be low. None of the community children had HIV-infection or a history of infection requiring medical attention in the 4 weeks prior to enrollment.

There are a number of strengths in this study. We were able to combine two cohorts of healthy community children and children with severe malaria to assess normal renal function in a relatively large population of Ugandan children. As children were followed longitudinally we were able to use the child’s growth trajectory to identify height outliers. We were also able to assess AKI in a population of children with severe malaria from the same community as the healthy children. As the study included children from 6 months of age, we were able to evaluate methods to estimate bSCr during a period of physiological changes in GFR. All children were genotyped for sickle cell anemia and we were able to evaluate whether there were differences in SCr in community children with sickle cell trait, as there is evidence that sickle cell trait may lead to more subtle changes in kidney function [43] and increases risk of chronic kidney disease in some [44, 45], but not all populations [46]. Children with sickle cell disease have hyperfiltration [47–50] and increased tubular secretion of creatinine [47, 51] contributing to lower bSCr levels. Additional research is needed to understand how sickle cell trait and sickle cell disease modifies normal kidney function, affects bSCr estimation, and whether sickle cell anemia increases the risk of severe malaria-associated AKI in the population.

## Conclusion

AKI is an important complication in hospitalized children and is diagnosed based on a change in creatinine from known or estimated bSCr. As AKI in LMIC is often community-acquired, there is an urgent need to identify the most appropriate approach to estimate bSCr in low-income settings. We considered both height-dependent and height-independent age-based approaches to estimate bSCr, and conclude that the Pottel-height independent GFR estimating equation represents the most appropriate equation for Ugandan children and represents a promising approach to adapt to other LMIC settings.

## Supplementary information

**Additional file 1 Figure S1.** Creatinine-for-height and creatinine-for-age curves for healthy community children. **Table S1.** Distribution of AKI and AKI severity using different methods of estimating baseline SCr in severe malaria. **Table S2.** AKI prevalence (95% CI) in children with severe malaria based on age category.

## Data Availability

The datasets used and/or analyzed during the current study are available from the corresponding author on reasonable request.

## Abbreviations

AIC: Akaike Information Criterion
AKI: Acute Kidney Injury
BIC: Bayesian information criterion
BMI: Body mass index
bSCr: Baseline serum creatinine
CI: Confidence Interval
GFR: Glomerular filtration rate
HIV: Human immunodeficiency virus
KDIGO: Kidney Disease: Improving Global Outcomes
LMIC: Low- and middle income country
RR: Risk ratio
ROC: Receiver operator characteristic
WHO: World Health Organization
