# Supplementary material for: Methods to estimate baseline creatinine and define acute kidney injury in lean Ugandan children with severe malaria: a prospective cohort study
Source: BMC Nephrol. 2020 Sep 29;21:417. doi: 10.1186/s12882-020-02076-1 (PMC7526147; doi:10.1186/s12882-020-02076-1)
Supplement: Supplementary file 1 — Additional file 1 Figure S1. Creatinine-for-height and creatinine-for-age curves for healthy community children. Table S1. Distribution of AKI and AKI severity using different methods of estimating baseline SCr in severe malaria. Table S2. AKI prevalence (95% CI) in children with severe malaria based on age category. [file 12882_2020_2076_MOESM1_ESM.docx]

**Additional File 1**

Supplement to: Batte A, Starr MC, Schwarderer AL, Opoka RO, Namazzi R, Phelps Nishiguchi ES, Ssenkusu JM, John CC, Conroy AL. Methods to estimate baseline creatinine and define acute kidney injury in lean Ugandan children with severe malaria: a prospective cohort study.

**Supplementary Figures and Tables**

Figure S1. Creatinine-for-height and creatinine-for-age curves for healthy community children.

Table S1. Distribution of AKI and AKI severity using different methods of estimating baseline SCr in severe malaria

Table S2. AKI prevalence (95% CI) in children with severe malaria based on age category


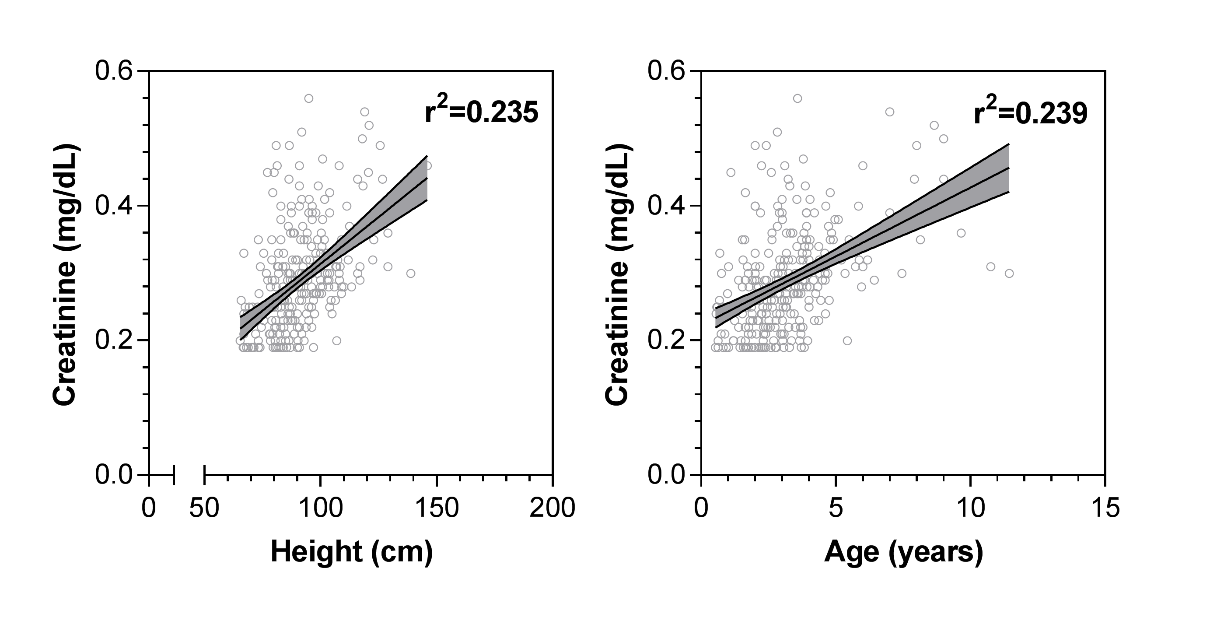


**Figure S1. Creatinine-for-height and creatinine-for-age curves for healthy community children.** We constructed creatinine-for-height and creatinine-for-age (height independent) curves from the community children to estimate baseline creatinine for the children with severe malaria.

**Table S1. Distribution of AKI and AKI severity using different methods of estimating baseline SCr in severe malaria**

|  | AKI %  (95% CI)^1^ | Stage 1 AKI %  (95% CI) | Stage 2 AKI %  (95% CI) | Stage 3 AKI %  (95% CI) |
| --- | --- | --- | --- | --- |
| AKI_Schwartz120_ | 31.4 (28.6, 34.2) | 17.3 (15.2, 19.7) | 8.0 (6.5, 9.8) | 6.1 (4.8, 7.7) |
| AKI_Pottel120_ | 40.4 (37.5, 43.3) | 22.8 (20.4, 25.4) | 10.6 (8.9, 12.6) | 7.0 (5.6, 8.6) |
| AKI _Schwartz137_ | 43.4 (40.4, 46.4) | 24.3 (21.8, 27.0) | 11.8 (10.0, 13.8) | 7.3 (5.9, 9.0) |
| AKI_upperlimit_ | 15.6 (13.5, 17.9) | 7.7 (6.2, 9.5) | 3.2 (2.3, 4.4) | 4.7 (3.6, 6.2) |
| AKI_heightCC_ | 39.2 (36.3, 42.2) | 22.4 (20.0, 24.9) | 10.1 (8.4, 12.1) | 6.8 (5.4, 8.4) |
| AKI_ageCC_ | 39.2 (36.3, 42.2) | 22.4 (20.0, 24.9) | 9.8 (8.2, 11.8) | 7.1 (5.7, 8.7) |

^1^Estimated using binomial exact CI

**Table S2. AKI prevalence (95% CI) in children with severe malaria based on age category**

|  | **<1 year**  **(n=74)** | **1 to <2 years**  **(n=296)** | **2 to <5 years (n=609)** | **Age ≥5 years (n=99)** |
| --- | --- | --- | --- | --- |
| AKI_Schwartz120_ | 37.8 (27.3, 49.6) | 37.3 (31.9, 43.0) | 28.9 (25.4, 32.6) | 24.2 (16.7, 33.8) |
| AKI_Pottel120_ | 47.3 (36.0, 58.9) | 45.6 (40.0, 51.3) | 38.4 (34.6, 42.4) | 31.3 (22.8, 41.3) |
| AKI _Schwartz137_ | 58.1 (46.4, 69.0) | 49.2 (43.5, 54.9) | 39.9 (36.1, 43.9) | 36.4 (27.4, 46.4) |
| AKI_upperlimit_ | 10.8 (5.4, 20.4) | 15.9 (12.1, 20.5) | 16.3 (13.5, 19.4) | 14.1 (8.5, 22.6) |
| AKI_heightCC_ | 45.9 (34.7, 57.6) | 43.7 (38.1, 49.5) | 36.8 (33.0, 40.7) | 35.4 (26.4, 45.4) |
| AKI_ageCC_ | 41.9 (31.0, 53.6) | 44.3 (38.7, 50.0) | 37.4 (33.7, 41.4) | 33.3 (24.6, 43.3) |

^1^Estimated using binomial exact CI
